# Dr. Wales on the Thousand Islands—Six Letters with Replies

**Published:** 1879-10

**Authors:** 


### Our Correspondence.

ALEXANDRIA BAY, Aug. 20, 1879.

The expedition was planned in the winter, and then it lay and mellowed until the following August. There were a few unimportant amendments from time to time; but for the main part it stood as first laid out.

The number concerned in it was not large, and when the advance was finally made, it consisted of only Bement and myself.

Our object of attack was an island lying beyond the jurisdiction of the United States—in fact within the Dominion of Canada, and occupied for the past seventeen years, by a gentleman by the name of Wallace. It was our intention to make this island our base of supplies, and a rendezvous on the return from our various incursions into the neighboring regions. Whether this was successfully carried out, remains to be told. But for the present, allow me a word or so, as to the line of our advance.

On the morning of the 6th of August, I lashed my gun case, and the wrappings of my fish rod firmly together, in order to reduce my baggage into the most compact form. I am not now about to launch into a disquisition on what you should carry on such an excursion, for personally, I carry just what I choose, and I've not the slightest intention of meddling with what any one else wishes to take.

However, I carried one thing too many, and that I'll tell about by and by, too. Our method of advancement toward the Canada line, was by the way of the U. I. & E. R. R., to Syracuse, and thence north by the R. W. & O. Road to Cape Vincent, then by steamer down the river to Alexandria Bay. From this point, if the double barrelled Custom House didn't capture us, to proceed by row boat to Wallace Island.

Of the journey up, let few words suffice. Only I wonder why we all call going north up. Of the dinner at a "Temperance House," of the persecutions endured at the hands of the "train boy," of the delusion and snare of taking a so-called palace coach from Syracuse; of the agony inflicted on us by a company of excursionists who had what they asserted to be a band, are not all these things buried deep in our hearts, and do you suppose we mean to dig them up again! Not at all.

But our railroad ride was not without a bright side, and among other things Bement made many new and curious discoveries about trees and birds, which served to keep up the hilarity of our minds.

At seven o'clock in the evening, we were at Cape Vincent, and changed to the deck of the "Island Belle" a little steamboat plying between the Cape and Alexandria Bay.

The asserted band took up a position in the after part of the upper deck, and we got as far forward as the limited length of boat allowed. But despite the distance and a head wind their painful strains still distressed our tortured ears. If the Iroquois Indians, who a couple of hundred years ago used to pursue this path on their way to make it lively for the French at Montreal, had had this so called band in their company, they would have been *facile princeps.* "One blast upon their bugle horns would have been worth a thousand men" (to paraphrase Scott,) for it would have driven every Frenchman on the river into fits, from the word go.

But no more of them. They did what they could.

The ride down the river was cold, and we were glad to wrap ourselves in overcoats and blankets. We saw nothing of interest but the ruins of the old French Fort on Long Island, and after a somewhat tedious stop at Clayton, we made a labored passage to the Bay. The entrance to Alexandria Bay was dramatic. The steamer swept around in a great curve, the two hotels burned Greek fire from their balconies, and amid the clash of martial music, and the howls of hotel porters we made our landing. The Crossman House gave us a good supper, and a clean bed and we fell asleep lulled, alas, even then, by the quadrille band in the parlor, giving forth for the light fantastic toe, the strains of Pinafore. "I'm called little Buttercup, dear little Buttercup, though I scarcely know why," wailed the music. "Well," said Bement, as he muffled his head in the bed clothes, "I'll be blamed if I do either!"

The next morning was clear and bright. Our oarsman was promptly on hand, and we soon started for Rockport, to interview the Custom House Officer, and announce our intention to intrench ourselves in Canada.

As soon as we were well out, over went the spoons, and we trolled for pickerel. I write pickerel intentionally, as all the time we were in Canadian waters, we waged a continual wordy warfare with the inhabitants of that country, who persist even to this day in calling them pike. But they *are* pickerel!

The writer of these pages added a peculiar lustre to his name by hooking a passing steamboat with his spoon, but failing to land the boat, he lost his spoon and about forty feet of rather poor line.

During the remainder of his stay among the Islands, he was referred to as the man who caught a steamboat, and no boat ever afterwards appeared but it was suggested he should attempt its capture. But none of these things moved him. He never tried to. On the way up from Rockport, Bement gallantly hauled in a hundred and fifty feet of line, and a big pickerel which weighed six pounds and a half, until we *weighed* him at Wallace Island, when he only weighed four and a half. And by the way, another season will find us wiser than we were this. We were so green as to *weigh* our fish. We have since found out that no "*true* sportsman" ever does that. For if a true sportsman did it, what would become of all the fifteen inch trout, the five pound bass, and the fifteen pound pickerel ? Mercy! They would fall away like water in a leaky tub. No, sir, next season we don't weigh our fish! We will leave that to greenies, boys, and pot-hunters.

Noon found us at Wallace Island, where we were hospitably received and famously fed before we unpacked our kit, got out our flies, and began legitimate work.

But it was not long before the strong arms of Mr. Wallace's son were impelling us toward the pet places, to hook the bold black bass.

Ah, it was sport! But to enjoy it fully, one must have had a good full year of hard work, and that had we. Seventeen nice bass rested in the box, when at dusk we wended our way back to the Island. Tea and cribbage brought an early bed time and we slept the sleep of honest men.

The waters of the Thousand Islands, abound in many fish. Sturgeon, muscalonge, pickerel, bass, perch, the ever present rock bass, the moon fish and chub. The chub to be taken there, would if caught in Chemung River, cause a war dance among the boys on Lake Street bridge that would be memorable. We took them frequently on our spoons that would *weigh on the scales* two pounds and a half, we returned them to the water.

Of the days that followed, I cannot write in detail. They were full of pleasure. Every night we returned with a full box of fish. We kept no bass that weighed less than half a pound, and took none that weighed over two pounds and a half. Our biggest pickerel weighed five and a half. The writer of this did not catch him; but he took a muscalonge that weighed *two pounds* (?)

We did not camp out, but slept comfortably in a good bed, in a clean house, and ate good food at a table, with knives and forks. Camping out is good fun when you must do it; but a tight roof, a cooking stove, and a spring bed suit me even when I have been fishing all day.

The Thousand Islands are not remarkable in our eyes for their beauty. They are interesting as a freak of nature, and there must be near two thousand of them. But they are too much alike. From one know all. Now and then we came upon a scene of rare beauty, but not often. We had a good time, and also went to Gananoque. Two weeks passed quickly, and then with brown backs to our hands and sunburned faces, we fell back in good order, into the United States.

THERON A. WALES.

---

CLEVELAND, O., Sept. 10, 1879.

DR. UP DE GRAFF:

Can I order your book BODINES of you, and does the $1.50 includes the postage on it ?

L. B.

Yes, or better still, order of the Publishers J. B. Lippincott & Co., Philadelphia, or of Hall Brothers, this city, who will supply all orders of this character.

---

MEMPHIS, TENN., May 29, 1879.

DR. UP DE GRAFF:

I have been a reader for some time of your "BISTOURY"—my father being an old subscriber. Seeing you kindly answer all enquiries, I write to know if you can recommend something to remove superfluous hair; have tried several advertised depilatories, but found none of value. I feel exceedingly anxious about it. The hair is on my arms and hands so as to disfigure and mortify me, and as I am a young lady with some pretentions to good looks, (excuse vanity), you will confer a great favor by helping me; and gain for yourself the best wishes and heart-felt thanks of,

Yours Respectfully,

MAMIE ——.

If your dress is properly constructed—with long sleeves, the hair upon your arms will not be seen, and will do you no harm. A few hours exercise in your mother's kitchen, daily, will remove the superfluous hair upon your hands. Try the dish pan and wash tub. They are excellent depilatories.

---

WASHINGTON, D. C., Aug. 12, 1879.

DR. UP DE GRAFF:

I was wounded during the war, through my elbow joint. It has been painful and has been suppurating, and particles of bone discharging ever since. Is there no way of curing it aside from amputation of the arm? J. D. N.

Consult some surgeon relative to resection. Perhaps the joint can be removed and the arm saved.

---

ST. LOUIS, MO., Aug. 1, 1879.

DEAR BISTOURY:

What a jolly companion you are! I would not be without you for ten times your cost. How I wish you would come monthly. Why don't you and charge us accordingly? That contributor of yours—Ben. H. Pratt, writes splendidly. So does Towner, and now and then Mr. Beecher puts in a word that always tells. You certainly manage to keep excellent help about you. Do you all write for the fun of it? I suspect so, for you really charge nothing for your journal.

Here's a dollar, and don't credit me for but one year, either. I consider the last BISTOURY worth ever penny of it.

Yours Gratefully,

J. R. D., M. D.

Thanks Doctor. We are not unmindful of praise, glad to know you like the BISTOURY. So do we, else would we not spend many precious moments over it.

---

BORDENTOWN, N. J., Sept. 1, 1879.

EDITOR BISTOURY:

I did not receive my last BISTOURY. Have taken it for years and it never failed to come before. Please send it to me. R.

You not only forgot to send your subscription, but neglected to send either price of the number wanted, or stamp to pay postage. We have so many applications of this sort that we are compelled to decline sending any back numbers, unless fifteen cents is enclosed in the order, to cover the expense.

HISEVILLE, Barren Co., KY., Jan. 22, 1879.

DR. THAD. S. UP DE GRAFF, Elmira, N. Y.

*My Dear Sir:*—Another year has passed away. My welcome friend, "THE BISTOURY" puts in his appearance, sprightly and instructive as ever.

He makes his bow, I take gladly his welcome hand, and feel strengthened with his cordial presence. "Come in, welcome guest," you shall have, as you deserve, a reserved seat in our family. Be my first right hand friend, and guest at table. When you want to ride, here's our best saddle horse, and when you wan't to take a drive, our best trotter is at your service. Our boys, two in number, five and three years of age, shall profit by your instructions, as soon as they can understand you.

I enclose subscription to "THE BISTOURY," for the ensuing year. May God bless *you* and *yours*, and may you prosper in all things pertaining to your future good and usefulness.

Very truly yours, T. S. N., M. D.

The BISTOURY approves of horse-back riding, and would be glad to try the best horse in our friend's stable, inasmuch as he resides in a country, famous for its superior saddle horses.

